# Diet Quality and Changes in Food Intake during the University Studies in Polish Female Young Adults: Linkages with Food Experiences from Childhood and Perceived Nutrition Concerns

**DOI:** 10.3390/nu14163399

**Published:** 2022-08-18

**Authors:** Patryk Pokorski, Robert Nicewicz, Marzena Jeżewska-Zychowicz

**Affiliations:** 1 Faculty of Human Nutrition, Warsaw University of Life Sciences (SGGW-WULS), Nowoursynowska 159C, 02-776 Warsaw, Poland; 2 Institute of Human Nutrition Sciences, Warsaw University of Life Sciences (SGGW-WULS), Nowoursynowska 159C, 02-776 Warsaw, Poland

**Keywords:** diet quality, food intake, parental feeding practices, childhood food experiences, nutrition concerns, young women, a cross-sectional study

## Abstract

The transition from adolescence to young adulthood may be associated with unfavorable changes in food intake due to some substantial transformations in social life and environment. Factors that affect food choices and diet quality during early adulthood are still not well-recognized. This paper aims to explore the relationship between females’ childhood food experiences related to parents’ monitoring practices and healthy eating guidance, perceived nutrition concerns, changes in food intake during university studies, and diet quality. A cross-sectional study with the use of CAWI (Computer-Assisted Web Interview) was carried out on 657 female students aged 19–30 years. Beliefs and Eating Habits Questionnaire (KomPAN) was used to assess the frequency of eating various kinds of food and then to calculate the diet-quality index (DQI). Adults’ Memories of Feeding in Childhood (AMoFiC) questionnaire was used to assess food experiences from childhood, and Health Concern Scale (HCS) was used to assess nutrition concerns. Associations between changes in food intake, diet quality, and selected factors describing the study sample were verified using logistic regression analysis. The DQI of the majority of students indicated a low intensity of nonhealthy and pro-healthy dietary characteristics. The intensity was higher among nutrition and health students, those with low nutrition concerns, and those with childhood experiences of monitoring. The chances of a negative change in food intake were increased by a higher score for childhood food experiences associated with parents’ monitoring practices. The chances of a negative DQI were increased by bigger concerns about nutrition and by technical and agricultural majors; however, they were reduced by positive changes in food intake. Both childhood food experiences and perceived nutrition concerns should be factored into strategies aimed at improving youth diet and educating parents about effective parental feeding practices.

## 1. Introduction

The transition from adolescence to young adulthood increases the risk of engaging in unhealthy food habits [1,2]. Although there is some evidence that dietary behaviors track from adolescence to adulthood [3,4], the transition from home to university life has been linked with unfavorable changes in food intake such as an increase in alcohol and sugar intake, consumption of sugar-sweetened beverages, calorie-dense snacks, and food away from home [5,6]. Moreover, young women tend to use various quantitative and qualitative dietary restrictions [7] that may lead to unhealthy dietary habits [8]. Young women’s eating habits are crucial for their health. In addition, as caregivers in the families, they play important roles in promoting healthy food intake and nutritional status of their family members [9]. In particular, maternal feeding practices have a crucial impact on the development of children’s eating habits [10]. The negative effect of changes in eating behaviors is poorer diet quality among emerging adults [11]. Diet modifications as well as body composition and weight gain are observed among young adults, including students [12,13]. However, there are still limited studies evaluating the stability of the comprehensive dietary indices (i.e., dietary patterns, overall diet quality) over time both from childhood to adolescence and from childhood to adulthood [5,14].

The transition from adolescence to young adulthood may be associated with increased autonomy over food choices, small food budgets, and exposure to new social groups and food cultures [15]. Moreover, it may include leaving home [16], changing or leaving school to begin further education or paid employment [17,18], and formation of partner relationships, including marriage, leading to cohabitation [19]. These changes are linked to dietary modifications [16]. Lipsky et al. [20] noticed that understanding diet-quality trajectories and identifying modifiable influences during this developmental stage is warranted given the initiation of many diseases. A better understanding of factors influencing food choices, diet quality, and dietary patterns during the age of early adulthood is necessary for the development and targeting of public health interventions. Less-recognized determinants of young people’s diets include childhood experiences and perceived nutrition concerns. 

The environment is of great importance in shaping human behavior [21,22], which is emphasized especially by radical behaviorists [23]. According to their theories, behavior is the result of the environment, with the environment being the external stimuli. Skinner argued that behavior can be understood by looking at one’s past and present environment and the reinforcements within it [24]. Strict behaviorists believe that all behaviors are the result of experience. Cognitive behaviorism, on the other hand, assumes that behavior is also influenced by thoughts and emotions. Despite criticism that behaviorism is a one-dimensional approach to understanding human behavior [23], it has many practical applications, such as in modifying problematic behavior and encouraging more positive behaviors. Both types of behaviorism, i.e., classical conditioning and operant conditioning, are methods that can be used by parents who are the gatekeepers of the early feeding environment. Parent–child interactions termed “food parenting” include feeding styles covering a wide range of feeding with the inclusion of emotions and parental feeding practices (PFP), which are specific strategies and actions (the “when, what, and how”) [25]. It has been shown that childhood experiences resulting from PFP are correlated with dietary practices [26,27] as well as with the food preferences of adults [28]. Such PFP as encouraging eating in a supportive manner, modeling favorable eating behaviors, and eating meals together might favor healthy eating behaviors and greater diet quality [29,30]. Since healthy dietary habits established during childhood and adolescence seem to be more stable over time than unhealthy ones [15], it can be assumed that the positive effects resulting from such feeding practices may persist well into early adulthood. However, there is still little research on the effect of parental feeding practices (PFP) on food intake among young, middle-aged, and older adults [31,32]. The retrospective study of Małachowska and Jezewska-Zychowicz [33] provided evidence that the intake of certain food groups in adulthood might be determined by food experiences resulting from parental feeding practices in childhood, which can incite researchers to further explore this relationship. In particular, it is crucial to develop new tools for assessing childhood experiences resulting from parenting practices and improve existing ones and, above all, to test their reliability by combining retrospective and long-term studies.

Several studies have found a positive relationship between perceived risk and health-related behaviors [11,34], while others have not found such a link [35]. According to the health belief model, the more serious the risk associated with a particular behavior of a person, the more open he or she is to any clues about that behavior, which should potentially trigger corrective action [36]. Therefore, it can be expected that higher perceptions of nutrition concerns will promote positive dietary changes, resulting in higher diet quality. For example, more frequent choice of organic food products due to the perceived negative consequences for human health related to pesticides [37,38] or stronger intentions to reduce red meat consumption due to health warning messages have been observed [39]. 

Some studies have suggested that healthy dietary habits are more stable over time than unhealthy ones [3,40,41]; hence, parental feeding practices conducive to the development of healthy eating behaviors as a source of childhood food experiences as well as perceived nutrition concerns were included in the study as explanatory factors for changes in food intake in early adulthood and diet quality. Since a relationship between these variables was assumed, the following hypotheses were formulated: (1) Having childhood food experiences related to parents’ monitoring practices and healthy eating guidance is associated with higher diet quality in early adulthood; (2) higher level of nutrition concerns is associated with higher diet quality in early adulthood; (3) having childhood food experiences related to parents’ monitoring practices and healthy eating guidance is associated with more positive changes in food intake during university studies; and (4) higher level of nutrition concerns is associated with more positive changes in food intake during the studying period. Thus, the purpose of the study was to explore the relationship between childhood food experiences related to parents’ monitoring practices and healthy eating guidance, perceived nutrition concerns, changes in food intake during the study period, and diet quality. 

## 2. Materials and Methods

### 2.1. Study Design and Sample Collection

A cross-sectional survey was conducted between January and May 2022. CAWI (Computer-Assisted Web Interview) technique was used to collect the data. Data confidentiality as well as anonymity were assured. The study was approved by the Ethics Committee of the Institute of Human Nutrition Sciences, Warsaw University of Life Sciences, in Poland (Resolution No. 29/2020). It was conducted in agreement with the guidelines of the Declaration of Helsinki. The sample was recruited from female students based on an advertisement on social media. The link for the questionnaire was posted in the announcement with a request for persons who meet the inclusion criteria to fill out the questionnaire. The following inclusion criteria were taken into account: women, Caucasian, Polish ethnicity, age of 18–30, being a student, and provided informed consent to participate. The study sample included 657 young women.

### 2.2. Dietary Data

The frequency of consumption of 24 food groups was assessed with the Beliefs and Eating Habits Questionnaire (KomPAN) [42], which was validated in Polish adults [43]. The participants reported the habitual frequency of eating food in the 3 months preceding the survey using one of the answers: 1—less than once a month or never; 2—1–3 times a month; 3—once a week; 4—a few times a week; 5—once a day; and 6—a few times a day. During the data analysis, the answers were converted to reflect the daily frequency of intake, ranging from 0—less than once a month or never; 0.06—1–3 times a month; 0.14—once a week; 0.5—a few times a week; 1—once a day; and 2—a few times a day [44].

“Diet-Quality Index” (DQI) was calculated based on 24 food groups, which included 10 groups with a potentially positive effect on health (pHDI), i.e., whole meal bread, wholemeal bread rolls, buckwheat, oats, wholegrain pasta, or other coarse-ground groats, milk, fermented milk beverages (e.g., yogurts, kefir), fresh cheese curd products (e.g., cottage cheese, homogenized cheese, fromage frais), white meat (e.g., chicken, turkey, rabbit), fish, pulse-based foods (e.g., from beans, peas, soybeans, lentils), fruit, and vegetables, and 14 groups with a potentially negative effect on health (nHDI), i.e., white bread and bakery products (e.g., wheat bread, toast bread, white bread rolls), white rice, white pasta, fine-ground groats (e.g., semolina, couscous), fast foods, fried foods (e.g., meat or flour-based foods such as dumplings, pancakes, etc.), butter (as a bread spread or as an addition to meals for frying, baking, etc.), lard, cheese (including processed cheese, and blue cheese), cold meats, smoked sausages, hot dogs, red meat (e.g., pork, beef, veal, mutton, lamb, game), sweets, tinned meats, sweetened carbonated or still beverages energy drinks, and alcoholic beverages. According to the procedure for calculating the QDI [44], the sum of the daily frequency of intake was calculated separately in both groups of foods, i.e., pHDI and nHDI. Then, the results for pHDI multiplied by 100/20 and for nHDI multiplied by 100/28 were added. For DQI, the range was from −100 to 100 points. A range from −100 to −26 points indicated a high intensity of nonhealthy dietary characteristics, from −25 to 25 indicated a low intensity of nonhealthy and healthy dietary characteristics, and from 26 to 100 indicated a high intensity of healthy dietary characteristics.

Changes in food intake during the study period were examined using the question “What is the difference between your current food intake and your food intake when you started studying?” using one of the answers: 1—“much higher intake”; 2—“slightly higher intake”; 3—“unchanged”; 4—“slightly lower intake”; and 5—much lower intake”. The question concerned the same 24 food groups, including 10 groups with a potentially positive effect on health (pHDI) and 14 groups with a potentially negative effect on health (nHDI). Unfavorable changes in diet, i.e., increased intake of nHDI foods, were coded as “−2”—“much higher intake” and “−1”—“slightly higher intake” and decreased intake of pHDI foods as “−1”—“slightly lower intake” and “−2”—“much lower intake”. In turn, favorable changes, i.e., increased intake of pHDI foods, were coded as “2”—“much higher intake” and “1”—“slightly higher intake”, while decreased intake of nHDI foods was coded as “1”—“slightly lower” and “2”—“much lower intake”. “Unchanged” was coded as “0”. An index describing the change in diet which occurred during the studies was then calculated, i.e., the Change in Food Intake (C-FI) index. It ranged from −48 to 48, where the higher the index, the more the changes improved the quality of the diet. 

### 2.3. Food Experiences from Childhood 

In the study, the Adults’ Memories of Feeding in Childhood (AMoFiC) questionnaire was used to enable retrospective reports of food experiences from childhood related to the application of certain parenting practices [33]. Respondents were asked to report how frequently different situations took place in their childhood, using a 6-point scale: 1– “never”; 2—“rarely”; 3—“sometimes”; 4—“mostly”; 5—“always”, and 6—“I don’t remember”. Moreover, they related to the sentences describing family habits from the period of their childhood using a 6-point scale: 1—“disagree”; 2—“slightly disagree”; 3—“neither agree nor disagree”; 4—“slightly agree”; 5—"agree”; and 6—“I don’t remember”. The answer “I don’t remember” was added to minimize the risk of recall errors. When calculating the score for the subscales within the AMoFiC, those who answered “I don’t remember” were excluded. Only 14 statements that form the two subscales healthy eating guidance (9 items) and monitoring (5 items) were used in the analyses. The overall score for each subscale was calculated by adding the scores for each item from the subscale. The higher the score, the more food experiences linked to parental feeding practices towards the respondent during childhood. Moreover, the mean value of the score for both subscales was calculated, and then, on its basis, two categories of study participants were identified: (1) people with a subscale score above the mean value defined as a “high” (presence of food experiences described by subscales) and (2) people with a subscale score equal to the mean value at most—defined as a “low” (lack of food experiences described by the subscales).

### 2.4. Perceived Nutrition Concerns

The selected statements from Health Concern Scale (HCS) [45] that relate to nutrition issues were used to assess participants’ concerns about nutrition, i.e., (1) I am concerned about consuming many calories; (2) I am concerned about consuming a lot of fat in my food; (3) I am concerned about consuming a lot of cholesterol in my food; (4) I am concerned about consuming a lot of sugar in my food; (5) I am concerned about consuming a lot of salt in my food; (6) I am concerned about providing a sufficient amount of energy with my food; and (7) I am concerned about food additives in my food. A 7-point scale starting from “definitely not” (1) through “neither no nor yes” (4) to “definitely yes” (7) was used to estimate each statement separately. The sum of the scores was calculated for the nutrition concerns index (NCI). The range for NCI is 7—49 points. Participants were divided into three groups based on the tertile distribution of NCI.

### 2.5. Sociodemographic Data

Questions on socio-demographic characteristics took into account: age (in years), place of residence before studying (village, town with less than 100,000 inhabitants, city with above 100,000 inhabitants), residence during studying (room in a dormitory or flat; rented flat; living with family), and study major. The study majors were categorized as (1) nutrition and health; (2) social and humanistic sciences; (3) psychology, and (4) other (technical and agricultural sciences). The body mass index (BMI) of the participants was calculated from self-reported body weight and height. The participants were categorized into four groups based on their BMI, according to the classification of the World Health Organization: underweight (BMI < 18.5 kg/m^2^), of normal weight (BMI between 18.5 and 24.9 kg/m^2^), overweight (BMI between 25.0 and 29.9 kg/m^2^), and obese (BMI ≥ 30.0 kg/m^2^). The last two categories were combined during data analysis as “overweight and obese”.

### 2.6. Statistical Analysis

Descriptive statistics were used to present the sociodemographic characteristics of the study sample. Data were presented as a sample percentage (%) for categorical data or mean and standard deviation (SD) for continuous data. The normality of the distribution of continuous variables was assessed with the normality Kolmogorov–Smirnov test, Lilliefors test, and normal probability plot. The independence χ2 test was used to assess the differences between groups identified according to sociodemographic characteristics. The Mann–Whitney U-tests and Kruskal–Wallis H-tests with adjustment by the Bonferroni correction were used to compare mean values. The internal consistency of items within subscales, i.e., Healthy Eating Guidance and Monitoring from the AMoFiC questionnaire and NCI were tested using Cronbach’s alpha, with values higher than 0.70 considered acceptable. Associations between changes in food intake during the study, current diet quality, and selected factors describing the study sample were verified using logistic regression analysis. The QDI and C-FI were treated as dichotomous variables (DQI ≤ 0 points, and DQI > 0 points; C-FI ≤ 0 points, and C-FI > 0 points) were dependent variables. The independent variables were: change in food intake—C-FI (continuous, points), healthy eating guidance-related food experiences (continuous, points), monitoring-related food experiences (continuous, points), concerns about nutrition (continuous, points), BMI (continuous, kg/m^2^), study major (categorical, nutrition and health/social and humanistic sciences/psychology/other), place of residence before studying (categorical, village/a town with less than 100,000 inhabitants/a city with over 100,000 inhabitants), and residence during studying (categorical, room in a dormitory or flat/rented flat/living with family). Odds ratios (ORs) represented the probability of adherence to those who reported the negative changes in food intake (C-FI ≤ 0) and displayed DQI ≤ 0, separately. The reference groups (OR = 1.00) were those who reported positive changes (C-FI > 0) and displayed DQI > 0, respectively. Wald’s test was used to assess the significance of ORs. *p*-value < 0.05 was considered significant for all tests. 

The analyses were carried out using IBM SPSS Statistics for Windows, version 28.0 (IBM Corp, Armonk, NY, USA).

## 3. Results

### 3.1. Characteristics of the Study Sample

Table 1 presents the sociodemographic characteristics of the participants. The sample consisted of 657 young women aged 19–30 years. Normal body weight characterized 73.8% of the study sample.

### 3.2. Food Experiences from Childhood

More people had healthy eating guidance-related food experiences (62.1%) than monitoring-related food experiences (47.9%). There were no differences in childhood food experiences (high/low) after taking into account study major, BMI category, or place of residence before studying and during the studying period. However, significant differences were found in the total score for the monitoring subscale after taking into account the place of residence before studying. A higher score was observed in the group of people living in a city with over 100,000 inhabitants compared to people living in a village. In addition, underweight subjects scored higher on the subscale healthy eating guidance than normal-weight subjects (Table 2).

### 3.3. Perceived Nutrition Concerns

The higher BMI individuals had, the more characterized they were by higher concerns about nutrition (Table 3). The highest concern about nutrition was reported by overweight and obese women (mean value 27.8 points) and the lowest by underweight ones (22.9 points). Study major, place of residence both during studies and before studies, but also food experiences from childhood resulting from parental feeding practices related to monitoring and healthy eating guidance did not differentiate the mean values of perceived concerns about nutrition in the study sample. Nearly 45% of underweight respondents displayed low concerns about nutrition, and almost as many overweight and obese participants (46.6%) were highly concerned about their nutrition. The fewest people living in rented flats during studies had moderate concerns about nutrition, while the fewest people living with their families had high concerns (Table 3).

### 3.4. Diet Quality and Changes in Food Intake during the Studies

Changes in food intake (C-FI) during the period of the study and diet quality index (DQI) of participants are presented in Table 4. Food intake changed more favorably in the normal-weight group compared to the overweight and obese ones, while there was no difference between the change in food intake in the underweight group compared to the others. In addition, there was a more positive change in food intake among those reporting low nutrition concerns compared to those with moderate nutrition concerns. The other variables included in the study did not significantly differentiate changes in food intake during the study period.

The DQI ranged from −45.6 to 58.0 points, which confirms the existence of irregularities in the diets of some students. Nevertheless, only 8 students were found to have a harmful effect of diet on their health (DQI low < −25), while 65 students (9.7%) were found to experience a beneficial effect of diet on their health (DQI > 25). Students of nutrition and health majors had higher dietary quality (DQI) compared to students of social and humanistic sciences and other faculties. In contrast, there were no differences in DQI among those studying nutrition and health sciences, and psychology. The latter had a higher DQI compared to students of technical and agricultural majors. Students with low nutrition concerns had the highest QDI, which was significantly superior than for both other groups, i.e., respondents with moderate and high nutrition concerns. There were no differences in QDI between those with moderate and high nutrition concerns. Subjects with childhood monitoring experiences had a higher QDI than others (Table 4).

More than half of the study group (51.8%) did not change their food intake adversely while studying, whereas no positive changes were made by 26.8% of the subjects. About one-fourth of the subjects only increased their intake of unfavorable foods (27.4%). One-third of the subjects (33.0%) made positive changes, which included both increasing the intake of favorable foods and decreasing the intake of unfavorable foods at the same time (Table 5). There were no differences in the occurrence of positive and negative changes during university studies after taking into account the characteristics of the study group, with the exception of negative changes, major study, and concerns about nutrition (Table 6). 

Among students from technical and agricultural majors, there was the largest number of people who increased the intake of unfavorable food and decreased the intake of favorable food (12.0%). This group also included the largest number of people who decreased their intake of favorable foods (17.4%) or increased their intake of unfavorable foods (30.4%). The least negative changes were made by those studying nutrition and health (60.7%) but also by those with low concern for nutrition (61.3%) (Table 6).

### 3.5. The Relationship between Changes in Food Intake during the Studies, Diet Quality, and Some Characteristics of the Study Sample

The relationship between changes in food intake during the studies and DQI was positive and moderate (0.407, *p* < 0.01). The results explaining the chances of negative change in food intake while studying and the negative diet quality index are presented in Table 7. Each point of increase in the change in food intake during the studies (C-FI score) reduced the chance of a negative DQI score by 12%. In contrast, a 1-point increase in the score for concerns about nutrition increased the chance of a negative DQI by 4%. In addition, students of technical and agricultural sciences were almost three times more likely to have a negative DQI score than students of nutrition and health majors (OR—2.88). Each 1-point increase in having monitoring related food experiences incremented the chance of a negative DQI by 5%. Healthy eating guidance-related food experiences, BMI, and place of residence before and during studying did not predict the chances of both negative C-FDI and negative QDI.

## 4. Discussion

Results of the study have shown that childhood food experiences related to parents’ monitoring practices and healthy eating guidance, perceived nutrition concerns, changes in food intake during the period of the study, and diet quality are interrelated. Furthermore, some participants’ characteristics, such as major of studies, place of residence during the studies, and BMI, differentiated the variables included in this study. It was found that positive changes in food intake while studying occurred, especially among respondents with normal body weight and low nutrition concerns. Changes in food consumption during university education correlated positively with DQI. The DQI of the majority of students indicated a low intensity of nonhealthy and pro-healthy dietary characteristics; however, it was higher among students of nutrition and health majors, those with low nutrition concerns, and those with childhood experiences of monitoring. 

### 4.1. Changes in Food Intake and Quality of the Diet in Early Adulthood

The occurrence of changes in food intake as a part of the transition from adolescence to young adulthood [1,46] was confirmed in our study. There were more positive changes observed than negative ones, which might have promoted improvements in the overall diet quality of young adults while studying. Among the positive changes, there were slightly more modifications that involved increasing the consumption of favorable foods compared to those reducing the consumption of unfavorable foods. Thus, our results confirmed that the transition to university life was linked with changes in the diet, which might result from increased autonomy over food choices, small food budgets, and exposure to new social groups and food cultures [15]. The overall index used to describe changes in food intake (C-FI) does not report specific changes but just a trend, which appeared to be positive. Previous studies investigating the changes in dietary habits of young people also found both an increased consumption of fried foods, sweet foods, and fruits but also vegetables and legumes [47,48]. Moreover, as the age increased, the eating habits became more regular and healthier [49,50]. Thus, the changes observed earlier do not have a consistent direction [16], which is in line with our results. Nevertheless, interpretation of the obtained results should be cautious. First of all, the results concern a group of young women, who are characterized by higher levels of functional nutrition literacy than men [51]. In addition, they are also more likely than men to maintain energy balance [52], have greater culinary skills, and prefer a “vegetarian” dietary pattern [2]. 

In the study group, about three-fourths of the respondents made positive changes to their diet, while half of them made negative ones. Positive changes included both an increase in consumption of favorable foods and a decrease in consumption of unfavorable foods (33%). Only 10% of the respondents reported negative changes in their diet. As it turned out that improvements in the diet were achieved in different ways among young people, it seems both types of modifications are worth encouraging and not solely the reduction of the consumption of foods that are detrimental to health. This is particularly true since the results of other studies show an increase in the consumption of fast food, EDNP snacks, and meat as well as a reduced intake of fruits, vegetables, and whole wheat cereal products [53,54]. 

In our study, the declared changes in the diets indicated their improvements; however, the results of other studies showed that the overall diet quality of European students became lower during their time spent at the university [55,56]. Moreover, the diet quality index was worse among younger people and men [57]. In our study, the majority had a DQI indicating a neutral effect of diet on health (range from −25 to 25 points) [41]. The neutral effect of diet on health was also observed in another study on young Poles [12]. An increase in the score for C-FI reduced the chances of a negative DQI score and therefore counteracted the deterioration of the diet. Thus, the hypothesis indicating that the changes in food intake during university studies are associated with lower diet quality in early adulthood was not verified. However, there is still a need for research in this area that would include men but also different approaches to data analysis. In addition, the results of our own study indicate that individual characteristics of young people may show a relationship with changes in food consumption during the course of their studies; thus, they need to be focused on in further research as mediating factors.

A variation in changes in food intake over the study period was observed after accounting for BMI and concerns about nutrition. More positive changes in food intake were noted especially in the normal-weight group compared to the overweight one but also among those reporting low nutrition concerns compared to those moderately concerned. It can be assumed that self-reported changes in food intake and concerns about nutrition partly explain the fact that those with low concerns for nutrition made fewer negative changes than those with moderate concerns. The group with high nutrition concerns necessitates further research that would explain why awareness of dietary errors (one of the reasons for being highly concerned about nutrition) is not sufficient to make changes to correct the current diet. 

### 4.2. Relationship between Childhood Food Experiences and Diet in Early Adulthood

In the study sample, more people had food experiences resulting from parental feeding practices, such as making healthy foods available, modeling the consumption of healthy food, and participating in the process of choosing, buying, and preparing meals (healthy eating guidance) than from practices of monitoring children’s eating behavior. At the same time, there was no variation in having such experiences according to the major of studies, BMI, or place of residence before studying and during the studying period. This may indicate the use of similar parental feeding practices toward children in the general population. However, the retrospective nature of the study might have also meant that variation in these practices during childhood was not reflected in respondents’ memories during adulthood. A study by [58] demonstrated the differences in parent feeding practices according to diverse socioeconomic, anthropometric, and behavioral correlates. Such differences were observed in our study in the mean values obtained for both groups of feeding practices (subscales). A higher total mean score for the “Monitoring” subscale was observed in the group of people living in a city with over 100,000 inhabitants compared to people living in the countryside. In addition, underweight people scored higher on the subscale healthy eating guidance than subjects characterized by normal weight.

Having food experiences related to parents’ monitoring practices applied in childhood was associated with higher diet quality in early adulthood. These results indicate that paying attention to what a child eats (sweets, snacks products rich in fat, sweetened beverages) may promote a better diet in adulthood due to the development of an awareness of the need to regulate the diet. It can also be speculated that monitoring practices may foster the ability to monitor one’s own behavior in adulthood. Previous studies on the relationships between parental monitoring and children’s diet have produced, however, inconsistent findings. Some cross-sectional studies suggested that parental monitoring is associated with a healthier diet (e.g., more fruit, vegetables, and fiber and fewer snacks, sweets, and sugary beverages) [59,60]. A positive health effect was also shown in a prospective observational study in which monitoring a child’s intake of high energy-dense foods at age 2 was associated with a healthier weight status one year later [61]. However, there are also studies in which associations between monitoring and children’s diet or weight were not observed [62,63]. Inconsistent results may result from a curvilinear relationship between parental monitoring and children’s dietary behavior. Monitoring may promote a healthy diet to a certain point, but too much monitoring may be counterproductive and may affect dietary behavior in a negative way [64]. Thus, the optimum level of monitoring may depend on the child’s individual characteristics. Some children may require more parental control than others [65]. Monitoring of child’s behavior was correlated with higher consumption of fresh fruits and vegetables, which also has a later reference in adulthood [29]. In the study sample, having monitoring experiences was associated with higher DQI; however, it did not differentiate the C-FI, which contradicted our hypothesis about the relationship between those experiences and more positive changes in food intake while studying. Previous findings showed that according to students’ opinions, their eating behaviors were shaped by their individual preferences and the social and environmental factors at university [66]. Moreover, they indicated that a lack of parental control affected their eating behavior while studying [67].

The lack of association between the DQI in adulthood and the experiences of PFP that foster beneficial eating behaviors (healthy eating guidance) is difficult to explain. It can be expected that more healthful eating behaviors formed in childhood through such practices [68] would also have a beneficial effect in adulthood. Research conducted among children suggests that PFP such as encouraging eating in a supportive manner, modeling favorable eating behaviors, eating meals together, and allowing children to participate in the process of choosing, buying, and preparing meals may favor healthy eating behaviors in children, including higher intake of fruit and vegetables [29,58] and greater diet quality [69]. Similarly, monitoring turned out to be associated with higher consumption of fresh fruits and vegetables [70]. On the other hand, previous retrospective studies conducted in adults taking into account the consumption of certain foods confirmed our results. It has been shown that intake of sweets and salty snacks in adulthood was associated neither with practices within healthy eating guidance nor within the monitoring subscale [33]. In contrast, a higher score for monitoring and healthy eating guidance was associated with a high fruit and vegetable intake in the group of young adults [33], which was also found in studies conducted among children [58,68]. The use of a more general indicator, i.e., the DQI, in our study may explain these differences.

Scores for both monitoring and healthy eating guidance subscales were not predictors of diet quality (DQI) in early adulthood (Table 7). In contrast, the higher was the score for experiences resulting from parents’ monitoring practices, the more the chances of negative C-FI increased. This result may indicate that people with such childhood experiences can experience a need in adulthood to limit monitoring of the intake of foods that adversely affect the diet. Arguably, as in the case of limited foods, the forbidden fruit effect may appear, leading to excessive food preoccupation. The desire to eat those products (limited or monitored in childhood) in adulthood can lead to higher consumption of some foods [71]. Moreover, monitoring can in certain situations develop into coercive practices, as can restraint practices, which may promote the forbidden fruit effect [72]. It seems that the results obtained regarding the relationship between the two types of parental feeding practices and the DQI should be considered in a more general context. PFP can be organized into three higher-order constructs [25], i.e., coercive control, structure, and autonomy-support or -promotion practices. Thus, the inclusion of selected practices representing the structure category may explain the lack of expected results in relation to the overall dietary index. In addition, some of the practices associated with monitoring might have become coercive, which could have negatively impacted the overall diet index in adulthood [73,74]. Further research is needed in order to identify the relationship between the DQI and different groups of PFP more comprehensively.

### 4.3. Relationship between Diet in Early Adulthood and Other Variables

Students with low nutrition concerns were characterized by a higher DQI score than those declaring moderate nutrition concerns, and most of respondents with low nutrition concerns did not declare negative changes. Furthermore, each point of increase of the nutrition concerns augmented the chances of a negative DQI by 4%. Thus, the hypothesis that a higher level of nutrition concerns is associated with a better diet quality in early adulthood was not confirmed. Low nutrition concerns may stem from a low awareness among young people about the nutrition recommendations [75], which may determine unhealthy eating. However, our results indicate that an inverse relationship should also be considered, that is, that healthy eating behaviors do not cause concerns about nutrition. Thus, low concern about nutrition can result from both low nutritional awareness but also from high nutritional awareness accompanied by healthy eating behaviors. It is possible that as students mature, they become increasingly aware of the impact of dietary choices on health and well-being and thus consider health an increasingly important determinant of food choices [2]. In our study, similarly to other studies [76,77], students of nutrition and health majors had higher dietary quality (DQI) compared to students of social and humanistic sciences and from other faculties. Despite students’ vast nutritional knowledge of nutrition and health (not assessed in this study), which may explain the higher DQI [12], there were no differences in nutrition concerns between students of different majors. The results of previous studies indicate more healthy behaviors of those with greater nutrition knowledge [78,79]. However, there is a need to clarify the relationship between nutrition knowledge, nutrition concerns, and eating behaviors, also measured by more comprehensive indicators such as dietary patterns or the DQI.

The positive relationship between BMI and perceived nutrition concerns in the study sample confirmed that nutrition concerns are unlikely to protect against negative behaviors but rather arise as a consequence of the latter. Hence, people who are overweight and obese, which is mostly a consequence of an inadequate diet, being aware of the mistakes they make, declared higher nutrition concerns. On the other hand, those with a more adequate diet declared fewer nutrition concerns. Previous studies showed a multidimensional association between concerns about nutrition and choice of food. They observed both negative correlations between nutrition concerns and intake of refined food and sweetened beverages but also a positive correlation with the intake of fast food [11] and fatty foods [34]. 

The transition from adolescence to young adulthood is often associated with a change of residence as well as with first attempts at running one’s own household. Research shows that adolescents who have left the family home during university often have poorer eating habits than they did before the studies [66,80]. The responsibilities linked with studying and independent living force students to become involved in food selection and to perceive the high prices but also the high availability of processed foods, which does not motivate them to prepare meals [81,82]. This may explain the increase in fast food consumption and other unhealthy behaviors [66,83]. In our study, female students living at home displayed better eating behaviors than those who lived in rented rooms [77]. Students living in dormitories consumed fruits and vegetables frequently and expressed intentions to eat healthily [83]. However, living in a dormitory was more often associated with alcohol consumption and eating late at night compared to living at home [84]. Living on academic campuses may be helpful for practicing healthy eating through access to appropriately balanced foods and beverages [85], but achieving this effect requires an implementation of policies and practices that support healthy eating and physical activity [86]. The study showed no differences in changes in food intake and in DQI after accounting for both residences before and during the study, which is consistent with the results of the study by [87]. The lack of differences may be linked to the female sample of respondents, who show more interest in a healthy diet also when living in a dormitory [83]. On the other hand, the place of residence during the study period differentiated the declared concern for nutrition. More people living outside the family home (both in a dormitory and rented room/flat) were characterized by either low or high concern for nutrition compared to those living in the family home. Thus, one can draw a conclusion that the availability of food but also the financial situation of the respondents may have played a role in conditioning these declarations. Further research should take this characteristic into account to explain the above-mentioned differences.

#### Limitation of the Study

The study group consisting only of female students was certainly a limitation of this study. Moreover, the study group was not representative of the population of Polish female students due to their voluntary participation in the study. Any generalizations are thus unjustified. The study used the Adult Memories of Childhood Nutrition Scale (AMoFiC), which is based on memories from childhood. The findings based on retrospective self-reports could have been biased, for example, by imprecise recollections or social-desirability bias [33]. Moreover, the questionnaire provided a possibility of frequent use of the “I don’t remember” response. Another limitation of the study was that we only referred to the influence of parents on eating behaviors and the quality of the diet in adulthood. The influence of the grandparents or other caregivers of the child was not taken into account. The data were collected among Polish young women; hence, the results cannot be generalized to other women’s populations due to the differences associated with ethnicity or socioeconomic status [88]. Nevertheless, this methodological approach can be implemented in other populations, which would allow for changes in food intake and their determinants to be viewed in a broader cultural context. Another limitation is the cross-sectional design of this study, which does not allow for finding a causal relationship. Last but not least, we used FFQs to collect dietary data, and it has been previously shown that self-reported data is often biased by overestimation of pro-healthy foods and underestimation of foods considered unhealthy [89]. Moreover, BMI was calculated based on self-reported data. 

## 5. Conclusions

The results of the study showed that female respondents made more health-beneficial than negative changes in food intake ones during their studies, with their diets reflecting low intensity of nonhealthy and pro-healthy dietary characteristics at the time of the survey. Positive changes consisted of both an increase in the consumption of healthy foods and a decrease in the consumption of unhealthy foods. The most negative changes characterized students from technical and agricultural majors. It was found that improvements in the diet were achieved in different ways among young women; therefore, both types of modifications are worth encouraging and not only the reduction of the consumption of unfavorable foods. 

Despite the predominance of positive changes, the DQI of the majority of students indicated a low intensity of nonhealthy and pro-healthy dietary characteristics, which was higher among students of nutrition and health majors, those with low nutrition concerns, and those with childhood experiences of monitoring. When eating behaviors in early adulthood are predicted, childhood food experiences related to monitoring practices seem to be of great importance. On the one hand, they were associated with a higher DQI, but on the other, they also increased the chances of negative changes in food intake during the study period. However, understanding the mechanism of their effect on eating behavior in early adulthood requires further research with the inclusion of young men.

The chances of an occurrence of negative DQI were increased by higher concerns about nutrition and by studying technical and agricultural majors. Further research is needed to clarify these relationships, which should include nutrition awareness as a potential explanatory factor. The obtained results on the relationship between monitoring experiences and perceived concerns about nutrition but also residence during studying, major of studies, BMI, and diet in the transition to adulthood explain changes in food intake. 

Both childhood food experiences and perceived nutrition concerns were associated with the DQI and the changes made during the studies. The results are not completely consistent with existing knowledge; thus, further research is required using other tools but also in other study samples. Nevertheless, the obtained results indicate that childhood food experiences and perceived nutrition concerns should be factored into strategies aimed at improving youth diet and educating parents about effective parental feeding practices.

## Figures and Tables

**Table 1:** Characteristics of the study sample.

	Total	
	N *	%
Total	657	100.0
Study major		
Nutrition and Health	122	18.6
Social and Humanistic Sciences	278	42.3
Psychology	165	25.1
Other (Technical and agricultural sciences)	92	14.0
Place of residence before studying		
A village	215	32.8
A town with less than 100,000 inhabitants	202	30.8
A city with over 100,000 inhabitants	240	36.4
Residence during studies		
Room in a dormitory or flat	211	32.2
Rented flat	223	33.9
Living with family	223	33.9
BMI (kg/m^2^)		
Underweight (BMI < 18.5)	56	8.5
Normal weight (BMI 18.5–24.9)	485	73.8
Overweight (BMI 25–29.9)	85	12.9
Obese (BMI ≥ 30)	31	4.8
Age (mean; SD in years) (range)	23.2; 2.3 (19–30)	
BMI (mean; SD in kg/m^2^) (range)	22.5; 3.6 (16.3–40.9)	

* N, number of participants.

**Table 2:** Characteristics of food experiences from childhood in the study sample.

Items	Food Experiences Related to:					
	Monitoring (N * = 502) (Mean; SD *) (Range 5–25)	Healthy Eating Guidance (N = 459) (Mean; SD) (Range 9–45)	Monitoring * (N = 502)		Healthy Eating Guidance * (N = 459)	
			High	Low	High	Low
Total	15.1; 4.88	29.3; 7.27	47.9	52.6	62.1	37.9
Study major				*p* > 0.05 *	*p* > 0.05 *	
Nutrition and Health	14.7 ^a^; 4.74	29.7 ^a^; 7.40	44.3	55.7	62.1	37.9
Social and Humanistic Sciences	14.7 ^b^; 4.91	28.8 ^b^; 7.22	44.0	56.0	59.1	40.9
Psychology	15.9 ^c^; 4.76	30.2 ^c^; 7.71	54.5	45.5	66.4	33.6
Other (Technical and agricultural sciences)	15.1 ^d^; 4.88	29.3 ^d^; 7.27	48.5	51.5	62.5	37.5
Place of residence before studying			*p* > 0.05		*p* > 0.05	
A village	14.4 ^a^; 4.80	28.9 ^a^; 7.51	43.7	56.3	57.8	42.2
A town with less than 100,000 inhabitants	15.1 ^b^; 4.66	29.2 ^b^; 7.64	43.3	56.7	61.2	38.8
A city with over 100,000 inhabitants	15.8 ^a^; 5.06	29.9 ^c^; 6.67	54.0	46.0	66.9	33.1
Residence during studies (points)			*p* > 0.05		*p* > 0.05	
Room in a dormitory or flat	15.0 ^a^; 4.92	29.3 ^a^; 7.02	47.1	52.9	64.8	35.2
Rented flat	15.6 ^b^; 4.87	29.3 ^b^; 7.74	53.0	47.0	60.9	39.1
Living with family	14.7 ^b^; 4.84	29.3 ^c^; 7.06	42.1	57.9	60.4	39.6
BMI (kg/m^2^)			*p* > 0.05		*p* > 0.05	
Underweight (BMI < 18.5)	16.2 ^a^; 4.76	32.2 ^a^; 6.24	56.1	43.9	74.4	25.6
Normal weight (BMI 18.5–24.9)	15.1 ^b^; 4.80	28.9 ^a^; 7.24	45.8	54.2	60.0	40.0
Overweight and obese (BMI ≥ 25)	15.0 ^c^; 5.26	29.5 ^b^; 7.59	50.0	40.0	64.7	35.3

* Chi^2^ test; ^a,b,c,d^—the same letters indicate statistically significant differences at *p* < 0.05 (Kruskal–Wallis H-test with adjustment by the Bonferroni correction). N, numbers of participants; SD, standard deviation.

**Table 3:** Perceived nutrition concerns in the study sample (N = 657).

Items	Concerns about Nutrition (Mean; SD) (Range 7–49)	Concerns about Nutrition *		
		Low (7–21)	Moderate (22–29)	High (30–49)
Total	25.4; 8.25	35.8	32	32.2
Study Major ** *p* > 0.05 *				
Nutrition and Health	24.1 ^a^; 8.36	43.5	29.5	27.0
Social and Humanistic Sciences	25.5 ^b^; 8.45	33.8	32.7	33.5
Psychology	25.6 ^c^; 8.35	35.2	31.5	33.3
Other (Technical and agricultural sciences)	26.4 ^d^; 7.13	32.6	33.7	33.7
Place of residence before studying ** *p* > 0.05				
Village	25.8 ^a^; 8.27	31.6	35.8	32.6
A town with less than 100,000 inhabitants	25.3 ^b^; 8.14	36.6	30.2	33.2
A city with over 100,000 inhabitants	25.2 ^c^; 8.34	38.8	30.0	31.2
Residence during studies ** *p* = 0.018				
Room in a dormitory or flat	25.2 ^a^; 7.92	35.5	33.6	30.9
Rented flat	25.4 ^b^; 8.36	39	23.8	37.2
Living with family	25.6 ^c^; 8.47	32.7	38.6	28.7
BMI ** (kg/m^2^) *p* = 0.004				
Underweight (BMI < 18.5)	22.9 ^a^; 8.18	44.6	26.8	28.6
Normal weight (BMI 18.5–24.9)	25.1 ^b^; 8.14	37.1	33.6	29.3
Overweight and obese (BMI ≥ 25)	27.8 ^ab^; 8.24	25.8	27.6	46.6
Monitoring *** (N = 502) *p* > 0.05				
High (above the mean score)	25.8 ^a^; 8.18	32.0	34.0	34.0
Low (below the mean score)	25.3 ^b^; 8.34	39.1	29.5	31.4
Healthy Eating Guidance *** (N = 459) *p* > 0.05				
High (above the mean score)	24.9 ^a^; 8.23	39.6	30.9	29.5
Low (below the mean score)	25.9 ^b^; 8.71	34.5	29.9	35.6

* Chi^2^ test; ** ^a,b,c,d^—the same letters indicate statistically significant differences; *p* < 0.05—Kruskal–Wallis H-test with adjustment by the Bonferroni correction; *** ^a,b^—the same letters indicate statistically significant differences; Mann–Whitney U-test, *p* < 0.05; SD, standard deviation.

**Table 4:** Change in food intake during the period of the study and Diet Quality Index in the study sample (N = 657).

Items	Change in Food Intake C-FI (Range—23–29)	Diet Quality Index DQI (Range—45.6–58.0)
Total	2.9; 6.56	6.1; 14.21
Study major *		
Nutrition and Health	3.9 ^a^; 7.31	10.0 ^ab^; 15.80
Social and Humanistic Sciences	2.7 ^b^; 6.57	4.5 ^a^; 13.85
Psychology	3.0 ^c^; 6.30	7.8 ^c^; 13.05
Other (Technical and agricultural sciences)	1.7 ^d^; 5.77	2.8 ^bc^; 13.60
Place of residence before studying *		
A village	3.1 ^a^; 6.06	5.5 ^a^; 13.50
A town with less than 100,000 inhabitants	2.6 ^b^; 6.94	6.1 ^b^; 15.07
A city with over 100,000 inhabitants	2.9 ^c^; 6.67	6.6 ^c^; 14.12
Residence during studying *		
Room in a dormitory or flat	2.3 ^a^; 6.79	4.3 ^a^; 14.51
Rented flat	3.3 ^b^; 6.93	7.3 ^b^; 13.78
Living with family	2.9 ^c^; 5.91	6.2 ^c^; 14.21
BMI * (kg/m^2^)		
Underweight (BMI < 18.5)	2.5 ^b^; 6.30	2.8 ^a^; 14.12
Normal weight (BMI 18.5–24.9)	3.2 ^a^; 6.49	6.8 ^b^; 13.96
Overweight and obese (BMI ≥ 25)	1.6 ^a^; 6.85	4.6 ^c^; 15.02
Concerns about nutrition *		
Low	3.9 ^a^; 6.60	9.2 ^ab^; 14.76
Moderate	2.0 ^a^; 6.13	3.6 ^a^; 12.23
High	2.6 ^b^; 6.80	5.1 ^b^; 14.84
Monitoring ** (N = 502)	3.0; 6.93	6.5; 13.99
High (above the mean score)	2.7 ^a^; 6.69	7.9 ^a^; 13.23
Low (below the mean score)	3.2 ^b^; 7.15	5.2 ^a^; 14.56
Healthy Eating Guidance ** (N = 459)	2.9; 6.81	7.0; 14.30
High (above the mean score)	3.0 ^a^; 6.48	7.7 ^a^; 13.91
Low (below the mean score)	2.8 ^b^; 6.81	5.8 ^b^; 14.87

* ^a,b,c,d^—the same letters indicate statistically significant differences, *p* < 0.05—Kruskal–Wallis H-test with adjustment by the Bonferroni correction; ** ^a,b^—the same letters indicate statistically significant differences; Mann–Whitney U-test, *p* < 0.05; SD, standard deviation.

**Table 5:** Positive and negative changes in food intake during the university studies (%).

Changes in:	Positive Changes in Food Intake (↑pHDI; ↓nHDI)		Negative Changes in Food Intake (↓pHDI; ↑nHDI)	
	%	N	%	N
pHDI and nHDI *	33.0	217	10.0	66
nHDI	14.2	93	27.4	180
pHDI	26.0	171	10.8	71
No positive/negative changes	26.8	176	51.8	340
Total	100.0	657	100.0	657

* pHDI, foods with a potentially positive effect on health; nHDI, foods with a potentially negative effect on health; **↑**, increase in the index; **↓**, decrease in the index.

**Table 6:** Negative changes in food intake during university studies according to study major and concerns about nutrition (%).

Variables	Negative Changes in:			
	↓pHDI and ↑nHDI *	↑nHDI	↓pHDI	No Negative Changes
Study major (*p* = 0.041)				
Nutrition and Health	9.8	19.7	9.8	60.7
Social and Humanistic Sciences	9.7	27.0	7.9	55.4
Psychology	9.7	32.1	12.7	45.5
Other (Technical and agricultural sciences)	12.0	30.4	17.4	40.2
Concerns about nutrition (*p* = 0.003)				
Low	8.9	22.1	7.7	61.3
Moderate	12.4	26.7	15.2	45.7
High	9.0	34.0	9.9	47.2
Total	10.0	27.4	10.8	51.8

* pHDI, foods with a potentially positive effect on health; nHDI, foods with a potentially negative effect on health; **↑**, increase in the index; **↓**, decrease in the index.

**Table 7:** Odds ratio (OR (95% CI) of change in food intake during the study period and Diet Quality Index for selected independent variables in the study sample.

Independent Variables	Change in Food Intake (C-FI)			Diet Quality Index (DQI)		
	Positive (C-FI >0)	Negative (C-FI ≤ 0) OR (95%CI)	*P* ^a^	DQI > 0	DQI ≤ 0 OR (95%CI)	*P* ^a^
Change in Food Intake (C-FI)				1	0.88 (0.85; 0.92)	<0.001
Healthy eating guidance-related food experiences	1	0.98 (0.95; 1.02)	0.331	1	0.97 (0.93; 1.00)	0.112
Monitoring-related food experiences	1	1.05 (1.00; 1.11)	0.041	1	1.00 (0.95; 1.06)	0.925
Concerns about nutrition	1	1.01 (0.98; 1.03)	0.537	1	1.04 (1.01; 1.07)	0.006
BMI	1	1.23 (0.81; 1.86)	0.335	1	0.71 (0.45; 1.12)	0.143
Study major (ref. nutrition and health)						
Social and Humanistic Sciences	1	1.41 (0.79; 2.53)	0.249	1	1.59 (0.83; 3.05)	0.161
Psychology	1	1.24 (0.67; 2.31)	0.493	1	1.34 (0.66; 2.69)	0.417
Other (Technical and agricultural sciences)	1	1.70 (0.82; 3.51)	0.155	1	2.88 (1.32; 6.31)	0.008
Place of residence before studying (ref. village)						
A town with less than 100,000 inhabitants	1	1.00 (0.60; 1.68)	0.999	1	1.25 (0.73; 2.16)	0.412
A city with over 100,000 inhabitants	1	1.00 (0.59; 1.69)	0.999	1	0.70 (0.40; 1.25)	0.233
Residence during studying (ref. room in a dormitory or flat)						
Rented flat	1	0.96 (0.58; 1.57)	0.856	1	0.92 (0.54; 1.57)	0.766
Living with family	1	1.27 (0.76; 2.11)	0.358	1	0.70 (0.54; 1.61)	0.801

^a^*P* significance (Wald’s test).

## Data Availability

The data presented in this study are available on request from the corresponding author.

## References

[1] Quaidoo E.Y., Ohemeng A., Amankwah-Poku M. (2018). Sources of Nutrition Information and Level of Nutrition Knowledge among Young Adults in the Accra Metropolis. BMC Public Health.

[2] Sprake E.F., Russell J.M., Cecil J.E., Cooper R.J., Grabowski P., Pourshahidi L.K., Barker M.E. (2018). Dietary patterns of university students in the UK: A cross-sectional study. Nutr. J..

[3] Movassagh E.Z., Baxter-Jones A.D.G., Kontulainen S., Whiting S.J., Vatanparast H. (2017). Tracking Dietary Patterns over 20 Years from Childhood through Adolescence into Young Adulthood: The Saskatchewan Pediatric Bone Mineral Accrual Study. Nutrients.

[4] Lake A.A., Adamson A.J., Craigie A.M., Rugg-Gunn A.J., Mathers J.C. (2009). Tracking of dietary intake and factors associated with dietary change from early adolescence to adulthood: The ASH30 study. Obes. Facts.

[5] Jongenelis M.I., Morley B., Pratt I.S., Talati Z. (2020). Diet Quality in Children: A Function of Grandparents’ Feeding Practices?. Food Qual. Prefer..

[6] Gallo L.A., Gallo T.F., Young S.L., Moritz K.M., Akison L.K. (2020). The impact of isolation measures due to COVID-19 on energy intake and physical activity levels in Australian university students. Nutrients.

[7] Banfield E.C., Liu Y., Davis J.S., Chang S., Frazier-Wood A.C. (2016). Poor Adherence to US Dietary Guidelines for Children and Adolescents in the National Health and Nutrition Examination Survey Population. J. Acad. Nutr. Diet..

[8] Munt A.E., Partridge S.R., Allman-Farinelli M. (2017). The barriers and enablers of healthy eating among young adults: A missing piece of the obesity puzzle: A scoping review. Obes. Rev..

[9] Black R.E., Victora C.G., Walker S.P., Bhutta Z.A., Christian P., de Onis M., Ezzati M., Grantham-McGregor S., Katz J., Martorell R. (2013). Maternal and child undernutrition and overweight in low-income and middle-income countries. Lancet.

[10] Liu X., Zhou Q., Clarke K., Younger K.M., An M., Li Z., Tan Y., Kearney J.M. (2021). Maternal feeding practices and toddlers’ fruit and vegetable consumption: Results from the DIT-Coombe Hospital birth cohort in Ireland. Nutr. J..

[11] Drywień M.E., Hamulka J., Jezewska-Zychowicz M. (2021). Perceived Nutrition and Health Concerns: Do They Protect against Unhealthy Dietary Patterns in Polish Adults?. Nutrients.

[12] Jeżewska-Zychowicz M., Plichta M. (2022). Diet Quality, Dieting, Attitudes and Nutrition Knowledge: Their Relationship in Polish Young Adults—A Cross-Sectional Study. Int. J. Environ. Res. Public Health.

[13] Ramón-Arbués E., Granada-López J.-M., Martínez-Abadía B., Echániz-Serrano E., Antón-Solanas I., Jerue B.A. (2021). Factors Related to Diet Quality: A Cross-Sectional Study of 1055 University Students. Nutrients.

[14] Appannah G., Murray K., Trapp G., Dymock M., Oddy W.H., Ambrosini G.L. (2020). Dietary pattern trajectories across adolescence and early adulthood and their associations with childhood and parental factors. Am. J. Clin. Nutr..

[15] Zaborowicz K., Czarnocińska J., Galiński G., Kaźmierczak P., Górska K., Durczewski P. (2016). Evaluation of selected dietary behaviours of students according to gender and nutritional knowledge. Rocz. Państwowego Zakładu Hig..

[16] Winpenny E.M., van Sluijs E.M.F., White M., Klepp K.-I., Wold B., Lien N. (2018). Changes in Diet through Adolescence and Early Adulthood: Longitudinal Trajectories and Association with Key Life Transitions. Int. J. Behav. Nutr. Phys. Act..

[17] Monge-Rojas R., Vargas-Quesada R., Colón-Ramos U., Chinnock A. (2022). Dietary Intake and Sources of Added Sugars in Various Food Environments in Costa Rican Adolescents. Nutrients.

[18] Kotova M.B., Maksimov S.A., Drapkina O.M. (2022). Gender, Age, Family and Territorial Features of Dietary and Physical Activity Patterns in Russian Youths. Int. J. Environ. Res. Public Health.

[19] Wirsching D., Baer N.-R., Anton V., Schenk L. (2022). Dietary Concepts in the Dyad: Results from a Qualitative Study of Middle-Aged and Older Couples. Appetite.

[20] Lipsky L.M., Nansel T.R., Haynie D.L., Liu D., Li K., Pratt C.A., Iannotti R.J., Dempster K.W., Simons-Morton B. (2017). Diet quality of US adolescents during the transition to adulthood: Changes and predictors. Am. J. Clin. Nutr..

[21] Park S., Kang J.H., Lawrence R., Gittelsohn J. (2014). Environmental influences on youth eating habits: Insights from parents and teachers in South Korea. Ecol. Food Nutr..

[22] Gubbels J.S. (2020). Environmental Influences on Dietary Intake of Children and Adolescents. Nutrients.

[23] Overskeid G. (2018). Do We Need the Environment to Explain Operant Behavior?. Front. Psychol..

[24] Moore J. (2013). Methodological behaviorism from the standpoint of a radical behaviorist. Behav. Anal..

[25] Vaughn A.E., Ward D.S., Fisher J.O., Faith M.S., Hughes S.O., Kremers S.P., Musher-Eizenman D.R., O’Connor T.M., Patrick H., Power T.G. (2016). Fundamental constructs in food parenting practices: A content map to guide future research. Nutr. Rev..

[26] Tarro S., Lahdenperä M., Vahtera J., Pentti J., Lagström H. (2022). Parental feeding practices and child eating behavior in different socioeconomic neighborhoods and their association with childhood weight. The STEPS study. Health Place.

[27] Mazza M., Morseth M., Torheim L.E. (2022). Association between parental feeding practices and children’s dietary intake: A cross-sectional study in the Gardermoen Region, Norway. Food Nutr. Res..

[28] Wadhera D., Capaldi Phillips E.D., Wilkie L.M., Boggess M.M. (2015). Perceived recollection of frequent exposure to foods in childhood is associated with adulthood liking. Appetite.

[29] Warkentin S., Mais L., Latorre M., Carnell S., Taddei J. (2016). Validation of the comprehensive feeding practices questionnaire in parents of preschool children in Brazil. BMC Public Health.

[30] Yee A.Z., Lwin M.O., Ho S.S. (2017). The influence of parental practices on child promotive and preventive food consumption behaviors: A systematic review and meta-analysis. Int. J. Behav. Nutr. Phys. Act..

[31] López-Banet L., Miguélez Rosique J.A., Martínez-Carmona M., Ayuso Fernández G.E. (2022). Development of Food Competence in Early Childhood Education. Educ. Sci..

[32] Swindle T., Rutledge J.M., Zhang D., Martin J., Johnson S.L., Selig J.P., Yates A.M., Gaulden D.T., Curran G.M. (2022). De-Implementation of Detrimental Feeding Practices in Childcare: Mixed Methods Evaluation of Community Partner Selected Strategies. Nutrients.

[33] Małachowska A., Jeżewska-Zychowicz M. (2021). Does Examining the Childhood Food Experiences Help to Better Understand Food Choices in Adulthood?. Nutrients.

[34] Noureddine S., Metzger B. (2014). Do Health-Related Feared Possible Selves Motivate Healthy Eating?. Health Psychol. Res..

[35] Andersson P., Sjoberg R.L., Ohrvik J., Leppert J. (2009). The effects of family history and personal experiences of illness on the inclination to change health-related behaviour. Cent. Eur. J. Public Health.

[36] Rosenstock I. (1974). Health belief model and preventive health behavior. Health Educ. Q..

[37] Miftari I., Haas R., Meixner O., Imami D., Gjokaj E. (2022). Factors Influencing Consumer Attitudes towards Organic Food Products in a Transition Economy—Insights from Kosovo. Sustainability.

[38] Wojciechowska-Solis J., Kowalska A., Bieniek M., Ratajczyk M., Manning L. (2022). Comparison of the Purchasing Behaviour of Polish and United Kingdom Consumers in the Organic Food Market during the COVID-19 Pandemic. Int. J. Environ. Res. Public Health.

[39] Borland S.E., Robinson S.M., Crozier S.R., Inskip H.M., SWS Study Group (2008). Stability of dietary patterns in young women over a 2-year period. Eur. J. Clin. Nutr..

[40] Taillie L.S., Prestemon C.E., Hall M.G., Grummon A.H., Vesely A., Jaacks L.M. (2022). Developing health and environmental warning messages about red meat: An online experiment. PLoS ONE.

[41] Descarpentrie A., Saldanha-Gomes C., Guivarch C., Dargent-Molina P., de Lauzon-Guillain B., Plancoulaine S., Charles M.-A., Chia A., Chong M.F.F., Vandentorren S. (2021). Family Socioecological Correlates of Lifestyle Patterns in Early Childhood: A Cross-Sectional Study from the EDEN Mother–Child Cohort. Nutrients.

[42] Jezewska-Zychowicz M., Gawecki J., Wadolowska L., Czarnocinska J., Galinski G., Kollajtis-Dolowy A., Roszkowski W., Wawrzyniak A., Przybylowicz K., Krusinska B., Gawecki J. (2018). Dietary Habits and Nutrition Beliefs Questionnaire for People 15–65 Years Old, Version 1.1.—Interviewer Administered Questionnaire. Dietary Habits and Nutrition Beliefs Questionnaire and the Manual for Developing of Nutritional Data.

[43] Kowalkowska J., Wadolowska L., Czarnocinska J., Czlapka-Matyasik M., Galinski G., Jezewska-Zychowicz M., Bronkowska M., Dlugosz A., Loboda D., Wyka J. (2018). Reproducibility of a questionnaire for dietary habits, lifestyle and nutrition knowledge assessment (KomPAN) in Polish adolescents and adults. Nutrients.

[44] Wadolowska L., Stasiewicz B., Gawecki J. (2020). The manual for developing nutritional data from the KomPAN^®^ questionnaire. KomPAN^®^ Dietary Habits and Nutrition Beliefs Questionnaire and the Manual for Developing Nutritional Data.

[45] Kähkönen P., Tuorila H. (1999). Consumer responses to reduced and regular fat content in different products: Effects of gender, involvement and health concern. Food Qual. Prefer..

[46] Quick V., Wall M., Larson N., Haines J., Neumark-Sztainer D. (2013). Personal behavioral and socio-environmental predictors of overweight incidence in young adults: 10-yr longitudinal findings. Int. J. Behav. Nutr. Phys. Act..

[47] Ruiz-Roso M.B., de Carvalho Padilha P., Mantilla-Escalante D.C., Ulloa N., Brun P., Acevedo-Correa D., Arantes Ferreira Peres W., Martorell M., Aires M.T., de Oliveira Cardoso L. (2020). COVID-19 Confinement and Changes of Adolescent’s Dietary Trends in Italy, Spain, Chile, Colombia and Brazil. Nutrients.

[48] Scarmozzino F., Visioli F. (2020). COVID-19 and the Subsequent Lockdown Modified Dietary Habits of Almost Half the Population in an Italian Sample. Foods.

[49] Entrena-Durán F., Baldan-Lozanom H., Valdera-Gil J.-M. (2021). Students’ Knowledge of Healthy Food and Their Actual Eating Habits: A Case Study on the University of Granada (Spain). Front. Sustain. Food Syst..

[50] Verwey N.L., Jordaan J., Friedeburg A.M.W. (2021). Dietary intake of first- and third-year female dietetics students at a South African university. South Afr. J. Clin. Nutr..

[51] Svendsen K., Torheim L., Fjelberg V., Sorprud A., Narverud I., Retterstøl K., Telle-Hansen V. (2021). Gender differences in nutrition literacy levels among university students and employees: A descriptive study. J. Nutr. Sci..

[52] Pendergast F.J., Livingstone K.M., Worsley A., McNaughton S.A. (2016). Correlates of Meal Skipping in Young Adults: A Systematic Review. Int. J. Behav. Nutr. Phys. Act..

[53] Varela-Mato V., Cancela J.M., Ayan C., Martín V., Molina A. (2012). Lifestyle and Health among Spanish University Students: Differences by Gender and Academic Discipline. Int. J. Environ. Res. Public Health.

[54] Lesińska-Sawicka M., Pisarek E., Nagórska M. (2021). The Health Behaviours of Students from Selected Countries—A Comparative Study. Nurs. Rep..

[55] Chourdakis M., Tzellos T., Papazisis G., Toulis K., Kouvelas D. (2010). Eating Habits, Health Attitudes and Obesity Indices among Medical Students in Northern Greece. Appetite.

[56] Hilger J., Loerbroks A., Diehl K. (2017). Eating Behaviour of University Students in Germany: Dietary Intake, Barriers to Healthy Eating and Changes in Eating Behaviour since the Time of Matriculation. Appetite.

[57] Zarrin R., Ibiebele T.I., Marks G.C. (2013). Development and validity assessment of a diet quality index for Australians. Asia Pac. J. Clin.Nutr..

[58] Warkentin S., Mais L.A., Latorre M.D.R.D.O., Carnell S., de Aguiar Carrazedo Taddei J.A. (2018). Relationships between parent feeding behaviors and parent and child characteristics in Brazilian preschoolers: A cross-sectional study. BMC Public Health.

[59] McGowan L., Croker H., Wardle J., Cooke L.J. (2012). Environmental and individual determinants of core and non-core food and drink intake in preschool-aged children in the United Kingdom. Eur. J. Clin. Nutr..

[60] Gubbels J.S., Kremers S.P., Stafleu A., de Vries S.I., Goldbohm R.A., Dagnelie P.C., de Vries N.K., van Buuren S., Thijs C. (2011). Association between parenting practices and children’s dietary intake, activity behavior and development of body mass index: The KOALA Birth Cohort Study. Int. J. Behav. Nutr. Phys. Act..

[61] Rodgers R.F., Wertheim E.H., Damiano S.R., Gregg K.J., Paxton S.J. (2015). “Stop Eating Lollies and Do Lots of Sports”: A Prospective Qualitative Study of the Development of Children’s Awareness of Dietary Restraint and Exercise to Lose Weight. Int. J. Behav. Nutr. Phys. Act..

[62] Webber L., Hill C., Cooke L., Carnell S., Wardle J. (2010). Associations between child weight and maternal feeding styles are mediated by maternal perceptions and concerns. Eur. J. Clin. Nutr..

[63] Mellin A.E., Neumark-Sztainer D., Story M., Ireland M., Resnick M.D. (2002). Unhealthy behaviors and psychosocial difficulties among overweight adolescents: The potential impact of familial factors. J. Adolesc. Health.

[64] Haycraft E., Blissett J. (2012). Predictors of paternal and maternal controlling feeding practices with 2- to 5-year-old children. J. Nutr. Educ Behav..

[65] Sogari G., Velez-Argumedo C., Gómez M.I., Mora C. (2018). College Students and Eating Habits: A Study Using an Ecological Model for Healthy Behavior. Nutrients.

[66] Deliens T., Clarys P., De Bourdeaudhuij I., Deforche B. (2014). Determinants of eating behaviour in university students: A qualitative study using focus group discussions. BMC Public Health.

[67] Blissett J., Bennett C., Fogel A., Harris G., Higgs S. (2016). Parental modelling and prompting effects on acceptance of a novel fruit in 2–4-year-old children are dependent on children’s food responsiveness. Br. J. Nutr..

[68] Lopez N., Schembre S., Belcher B., O’Connor S., Maher J., Arbel R., Margolin G., Dunton G. (2018). Parenting styles, food-related parenting practices, and children’s healthy eating: A mediation analysis to examine relationships between parenting and child diet. Appetite.

[69] Tan C., Ruhl H., Chow C., Ellis L. (2016). Retrospective reports of parental feeding practices and emotional eating in adulthood: The role of food preoccupation. Appetite.

[70] Scaglioni S., De Cosmi V., Ciappolino V., Parazzini F., Brambilla P., Agostoni C. (2018). Factors ]=−0 900Influencing Children’s Eating Behaviours. Nutrients.

[71] Daniels L.A. (2019). Feeding Practices and Parenting: A Pathway to Child Health and Family Happiness. Ann. Nutr. Metab..

[72] Batsell R.W., Brown A., Ansfield M., Paschall G. (2002). “You Will Eat All of That!”: A retrospective analysis of forced consumption episodes. Appetite.

[73] Ellis J., Galloway A., Webb R., Martz D., Farrow C. (2016). Recollections of pressure to eat during childhood, but not picky eating, predict young adult eating behavior. Appetite.

[74] Mead B.R., Christiansen P., Davies J.A.C., Falagán N., Kourmpetli S., Liu L., Walsh L., Hardman C.A. (2021). Is Urban Growing of Fruit and Vegetables Associated with Better Diet Quality and What Mediates This Relationship? Evidence from a Cross-Sectional Survey. Appetite.

[75] Sun-Choung A. (2020). A Comparative Study on the Dietary Habits and Nutrition Intake Tendency on the University Students in the Northern Gyeonggi Region by Their Majors. J. Korean Culin. Soc..

[76] Chae M.-O. (2022). Investigation of Nutrition Knowledge, Eating Behaviors, and the Need for Integrated Programs to Improve Eating Behaviors According to the Residential Type of Female College Students. J. Digit. Converg..

[77] Abraham S., Noriega Brooke R., Shin J.Y. (2018). College students eating habits and knowledge of nutritional requirements. J. Nutr. Hum. Health.

[78] Belogianni K., Ooms A., Lykou A., Moir H.J., Belogianni K. (2021). Nutrition knowledge among university students in the UK: A cross-sectional study. Public Health Nutr..

[79] Harris K.M., Gordon-Larsen P., Chantala K., Udry J.R. (2006). Longitudinal trends in race/ethnic disparities in leading health indicators from adolescence to young adulthood. Arch. Pediatr. Adolesc. Med..

[80] Allom V., Mullan B. (2014). Maintaining healthy eating behaviour: Experiences and perceptions of young adults. Nutr. Food Sci..

[81] Menozzi D., Sogari G., Mora C. (2015). Explaining Vegetable Consumption among Young Adults: An Application of the Theory of Planned Behaviour. Nutrients.

[82] Bagordo F., Grassi T., Serio F., Idolo A., De Donno A. (2013). Dietary habits and health among university students living at or away from home in southern Italy. J. Food Nutr. Res..

[83] Solhi M., Shirzad M. (2016). Situation of fruits and vegetables consumption in dormitory female students based on the theory of planned behavior. J. Health Lit..

[84] Nelson M.C., Kocos R., Lytle L.A., Perry C.L. (2009). Understanding the Perceived Determinants of Weight-related Behaviors in Late Adolescence: A Qualitative Analysis among College Youth. J. Nutr. Educ. Behav..

[85] Martin S., McCormack L. (2022). Eating behaviors and the perceived nutrition environment among college students. J. Am. Coll. Health.

[86] Hatfield D.P., Sharma S., Bailey C.P., Bakun P., Hennessy E., Simon C., Economos C.D. (2022). Implementation of nutrition and physical activity-related policies and practices on college campuses participating in the Healthier Campus initiative. J. Am. Coll. Health.

[87] Syihab S.F., Sultoni K., Suherman A. (2019). Association between types of domicile and nutritional status of college students in Indonesia. Advances in Social Science. Educ. Humanit. Res..

[88] Galloway A., Farrow C., Martz D. (2010). Retrospective Reports of Child Feeding Practices, Current Eating Behaviors, and BMI in College Students. Obesity.

[89] Forrestal S.G. (2011). Energy intake misreporting among children and adolescents: A literature review. Matern. Child. Nutr..

